# Testing the Recruitment Frequency, Implementation Fidelity, and Feasibility of Outcomes of the Heart Failure Activity Coach Study (HEALTHY): Pilot Randomized Controlled Trial

**DOI:** 10.2196/62910

**Published:** 2025-01-08

**Authors:** Andreas Blomqvist, Maria Bäck, Leonie Klompstra, Anna Strömberg, Tiny Jaarsma

**Affiliations:** 1 Department of Health, Medicine and Caring Sciences Linköping University Linköping Sweden; 2 Department of Occupational Therapy and Physiotherapy Sahlgrenska University Hospital Gothenburg Sweden; 3 Department of Cardiology Linköping University Linköping Sweden

**Keywords:** heart failure, disease management, physical activity, sedentary, older adults, aging, mobile health, mHealth, feasibility, quality of life, digital health, smartphone

## Abstract

**Background:**

Heart failure (HF) is a common and deadly disease, precipitated by physical inactivity and sedentary behavior. Although the 1-year survival rate after the first diagnosis is high, physical inactivity and sedentary behavior are associated with increased mortality and negatively impact the health-related quality of life (HR-QoL).

**Objective:**

We tested the recruitment frequency, implementation fidelity, and feasibility of outcomes of the Activity Coach app that was developed using an existing mobile health (mHealth) tool, Optilogg, to support older adults with HF to be more physically active and less sedentary.

**Methods:**

In this pilot clinical randomized controlled trial (RCT), patients with HF who were already using Optilogg to enhance self-care behavior were recruited from 5 primary care health centers in Sweden. Participants were randomized to either have their mHealth tool updated with the Activity Coach app (intervention group) or a sham version (control group). The intervention duration was 12 weeks, and in weeks 1 and 12, the participants wore an accelerometer daily to objectively measure their physical activity. The HR-QoL was measured with the Kansas City Cardiomyopathy Questionnaire (KCCQ), and subjective goal attainment was assessed using goal attainment scaling. Baseline data were collected from the participants’ electronic health records (EHRs).

**Results:**

We found 67 eligible people using the mHealth tool, of which 30 (45%) initially agreed to participate, with 20 (30%) successfully enrolled and randomized to the control and intervention groups in a ratio of 1:1. The participants’ daily adherence to registering physical activity in the Activity Coach app was 69% (range 24%-97%), and their weekly adherence was 88% (range 58%-100%). The mean goal attainment score was –1.0 (SD 1.1) for the control group versus 0.6 (SD 0.6) for the intervention group (*P*=.001). The mean change in the overall HR-QoL summary score was –9 (SD 10) for the control group versus 3 (SD 13) in the intervention group (*P*=.027). There was a significant difference in the physical limitation scores between the control (mean 45, SD 27) and intervention (mean 71, SD 20) groups (*P*=.04). The average length of sedentary bouts increased by 27 minutes to 458 (SD 84) in the control group minutes and decreased by 0.70 minutes to 391 (SD 117) in the intervention group (*P*=.22). There was a nonsignificant increase in the mean light physical activity (LPA): 146 (SD 46) versus 207 (SD 80) minutes in the control and intervention groups, respectively (*P*=.07).

**Conclusions:**

The recruitment rate was lower than anticipated. An active recruitment process is advised if a future efficacy study is to be conducted. Adherence to the Activity Coach app was high, and it may be able to support older adults with HF in being physically active.

**Trial Registration:**

ClinicalTrials.gov NCT05235763; https://clinicaltrials.gov/study/NCT05235763

## Introduction

### Background

Heart failure (HF) is a clinical syndrome characterized by a reduced cardiac output leading to a deficit in meeting the metabolic needs of the cells [1]. It is a major cause of morbidity and mortality worldwide [2]. Physical inactivity and sedentary behavior adversely affect outcomes in patients with HF. Sedentary behavior is a state where no activities performed increase energy expenditure substantially above the resting level, typically sitting or lying down [3]. Physical inactivity can be defined as the nonachievement of targets according to the physical activity guidelines that are recommended for an individual [4]. Although the 1-year survival rate for patients after the first diagnosis of HF is high [5], physical inactivity is associated with additionally increased mortality them [6]. Similarly, sedentary behavior is also associated with increased mortality [7] and negatively impacts the health-related quality of life (HR-QoL) [8]. It is, therefore, desirable to support patients with HF to be more physically active and break their sedentary behavior. A tool aimed at doing this, called “Activity Coach,” has been developed as a separate module on an existing mobile health (mHealth) tool [9].

The Activity Coach app is aimed at supporting everyday physical activity and reducing sedentary behavior and should not be viewed as an exercise tool. Furthermore, it is primarily intended for the physically inactive population with HF and is planned to be tested for efficacy in that population [9].

The Medical Research Council (MRC) suggests that before conducting an appropriately powered efficacy study, enough piloting and feasibility work has to be performed to be confident that the intervention can be delivered as intended (fidelity) and that safe assumptions can be made about effect sizes and variability and about rates of recruitment and retention [10]. A pilot randomized controlled trial (RCT) is the best way to assess the feasibility of a large, expensive full-scale study [11], and hence, the objectives of a pilot RCT need to be different from those of a future efficacy study, including comments on the uncertainties to be investigated [12]. In accordance with the MRC [10] and the CONSORT (Consolidated Standards of Reporting Trials) guidelines for pilot RCTs [13] (Multimedia Appendix 1), we conducted a pilot RCT to evaluate a study design, with the main focus being the rate of recruitment. In addition, we sought to assess the fidelity of the intervention, as well as the plausibility of the proposed outcome measures, in terms of data quality and effect sizes.

### Objective

The objective of this pilot RCT was to test the study protocol of an intervention in terms of patient recruitment, intervention fidelity, and plausibility of outcomes. The research questions were as follows:

- Recruitment: What was the recruitment frequency and attrition rate in the target population?
- Implementation fidelity: To what extent was the intervention delivered as intended?
- Outcomes: How was the plausibility of outcomes in terms of data integrity and quality, and what limited efficacy was seen regarding the included outcome measures?

## Methods

### Trial Design and Participants

The Heart Failure Activity Coach Study (HEALTHY) was a single-blinded, parallel pilot clinical RCT. Since the Activity Coach app was developed on the platform of an already existing mHealth tool called Optilogg, only people equipped with a home-based self-care support device called Optilogg were eligible to participate. As all study participants regardless of study arm allocation would have Optilogg since before, this also eliminated the risk of the effects of Optilogg per se confounding the results. Participants also needed to be listed at a primary health care center.

The eligibility criteria for participation were age≥18 years, a confirmed HF diagnosis, using Optilogg (obtained from a participating HF clinic), being physically inactive based on a screening question, and having the ability to consent to participation. Eligible people were excluded if they did not consent to a home visit or to using an accelerometer, if they had a life expectancy shorter than 6 months, or if they were already participating in another study on physical activity.

#### Change to Trial Design After Commencement

After the initial slow recruitment of just 2 randomized participants after 3 months, an amendment was submitted to the ethical review board, the inclusion criteria were modified, and it was decided that all people willing to participate would be included regardless of their answer to the screening question on physical inactivity. This change was justified based on the fact that the Activity Coach app might have benefits for a more active population with HF, too. Furthermore, potential participants were now contacted by telephone instead of regular mail. In the *Ancillary Analyses* section, potential differences between participants as stratified by the screening question are reported.

#### Study Procedure

Eligible people were first contacted by regular mail, and those willing to participate would be asked to answer a single-item self-report question. This question has previously been evaluated as a screening tool for classifying respondents as being physically active or physically inactive, and the same methodology was used in this study to identify physically inactive people [14]. The question is item 9 in the validated the European Heart Failure Self-care Behaviour Scale, and the question is in the shape of a statement (“I exercise regularly”), which the respondent answers using an ordinal scale from 1 to 5, with 1 being “I completely agree” to 5 being “I don’t agree at all” [15]. In this study, responses from 3 to 5 were coded as physically inactive [14]. If a participant met the eligibility criteria, a home visit was scheduled. During the home visit, the participant signed an informed consent form, was randomized to the intervention or the control group, filled out baseline questionnaires, and received an accelerometer. Lastly, the Activity Coach app or the sham version was installed on their Optilogg mHealth tool.

The intervention period was 12 weeks, and during weeks 1 and 12 of the study, participants wore the accelerometer every day. At the end of the study the same questionnaires that were completed at the start of the study were filled out again. Clinical data, such as demographics, etiology, comorbidities, and pharmacological treatments, were collected from the participants’ electronic health records (EHRs) by the HF nurse at each respective primary health care center.

### Intervention and Control

#### The mHealth Tool Optilogg

Optilogg consists of a tablet computer wirelessly connected to a weight scale, and the user is encouraged to interact with it daily. Without the addition of the Activity Coach app, Optilogg has 3 main features: subjective and objective symptom tracking, interactive education about living with HF, and an automated flexible loop-diuretics regime. It has been shown to improve self-care behavior in people with HF [16,17].

#### The Activity Coach App Intervention

The Activity Coach app was added as a novel feature to the existing Optilogg, with the purpose of supporting people with HF to be physically active, and was developed using principles described by the MRC [9,10]. Therefore, in addition to existing Optilogg features, the mHealth tool included Activity Coach features as well. Every day, the user can, in an intuitive graphical user interface (GUI), enter how much time they have been physically active in increments of 10 minutes regardless of what physical activity means for them. This allows for a one-size-fits-all approach to accommodate heterogenous populations. The accumulated physical activity is logged and presented as trends in a separate tab. Each week, there is an on-screen summary of the past week’s logged activity, and the user can, if desired, set a goal for the upcoming week, although they can at any time turn off the goal-setting feature. The progress toward reaching the goal is illustrated in the trends tab. The Activity Coach app was designed to first create motivation to be physically active and then provide a means of tracking physical activity. Motivation is achieved by providing an educational tip a day for 1 week, aimed at establishing 3 beliefs: physical activity leads to certain outcomes (positive outcome expectancy), the person can perform physical activity (self-efficacy), and the outcomes are desirable to the person (goal congruence). More details on the underlying intervention theory and theoretical model, as well as the development process, have been published elsewhere [9]. The participants in the intervention group were instructed to try to track their physical activity in the Activity Coach app daily.

#### Control Version

As previously mentioned, the original Optilogg contains an education module that, in part, deals with physical activity and exercise, and this education module has been shown to increase knowledge about HF [17]. This could have been a possible confounder when trying to analyze the additional effects of the Activity Coach app. To mitigate this, all participants were recruited from existing Optilogg users. The intervention group had the Activity Coach app installed on their existing Optilogg system, and the control group had a sham version of the Activity Coach app installed instead. This slightly changed the appearance of the GUI compared to the original Optilogg prior to the study, such that instead of starting each day on the regular home-screen, the modified system started in the section of the education module dealing with physical activity, but none of the actual Activity Coach features were enabled. This led to an apparent change, also relating to physical activity, which would be immediately noted by the user.

### Research Question 1: Recruitment

The recruitment procedure metric to be evaluated was defined as the number of people who met the eligibility criteria who needed to be contacted and screened to reach a certain number of recruited and randomized participants. Monitoring attrition rates was also part of evaluating the study design. As described earlier, the study design was changed to also include participants who originally were intended to not be included, since they were defined as physically active. Hence, this outcome was reported as 2 numbers: one where the original screening was still used and the other where physically active people were also included.

### Research Question 2: Implementation Fidelity

Implementation fidelity was evaluated by analyzing how much the participants randomized to the intervention group actually used the Activity Coach app (eg, adherence, which is a common means of assessing intervention fidelity in physical activity interventions) [18]. Adherence was defined as the ratio of actual use to intended use [19], and since, no clear recommendation exists on how often the Activity Coach app or similar behavior change interventions relating to physical activity should be used, we reported daily and weekly adherence. Daily adherence was calculated as the number of days that the participant performed physical activity divided by the study duration in days (ie, 84). Weekly adherence was calculated as the number of weeks that the participant performed physical activity in 1 day or more divided by the number of weeks in the study (ie, 12). Furthermore, the Activity Coach app offers a goal-setting feature on a weekly basis. The usage of this feature was also examined.

### Research Question 3: Outcomes

The outcome measures studied were the subjective goal attainment score; the HR-QoL, measured with the Kansas City Cardiomyopathy Questionnaire (KCCQ); and objective physical activity, measured with an accelerometer.

#### Subjective Goal Attainment

For goal attainment, we relied on the methods proposed by Urach et al [20]. Upon enrollment, participants selected 2 goals from a list of 10 physical activity–related goals they wanted to achieve during the 12-week study. The list was based on a literature review of the effects of increased physical activity on people with HF. The 10 goals are listed in Table 1. Upon completing their participation in the study, each participant rated the 2 selected goals during follow-up, comparing the current status with the status when the study started. The rating was on a scale from –2 to 2, where –2 indicated worsening and 2 indicated improvement. A mean value of the scores of the 2 goals was then calculated and constituted the reported value, referred to as the goal attainment score.

**Table 1:** Prespecified goals for the subjective goal attainment outcome.

Goal number	Goal
1	Better sleep
2	Boosted daytime energy
3	Able to walk with ease without frequent pauses
4	Less physical pain
5	Feel better
6	Reduced shortness of breath
7	Less daytime fatigue
8	More energy to do things around the house
9	Increased desire and energy for social activities
10	Improved appetite

#### Health-Related Quality of Life

The Kansas City Cardiomyopathy Questionnaire (KCCQ) short version is a 12-item questionnaire used to assess the HR-QoL in people with cardiomyopathies, which outputs 4 subscale summary scores (physical limitation, symptom frequency, quality of life [QoL], and social limitation), as well as a total summary score [21]. The scores range from 0 to 100, where 100 represents the best-possible value for any given score. The questionnaire has been validated in the current population [22].

#### Objective Physical Activity

Sedentary behavior and physical inactivity were assessed objectively using an accelerometer [23]. The accelerometer used was the validated ActiGraph GT9X [24], and participants were instructed to wear it around the hip for 7 consecutive days. The accelerometer data were reported as the percentage sedentary time, the average length of sedentary bouts, and steps per day. Data were sampled as raw data (30 Hz), converted to 60-second time intervals (or epochs) in counts per minute for the vector magnitude, and analyzed using ActiLife software (v.6.13.4).

Certain criteria were applied to the accelerometer data acquired for it to be included in the analyses. The accelerometer had to be used during at least 4 of the 7 days, with a minimum wear time of 540 minutes/day. Periods with consecutive 0s for 60 minutes or more (with 2-minute tolerance) were interpreted as time of nonuse and excluded from further analyses.

### Sample Size

An appropriate sample size for a pilot should be based on the estimated effect size, where a large effect size warrants 15-30 participants, an intermediate effect size requires 25-35 participants [25], and a more general recommendation is a sample size of 12 per group [26]. In this study, the target sample size was set to 40, and enrollment was to stop either when 40 study participants had been randomized or when all eligible people had been contacted (whichever came first).

### Randomization

Sheets of paper with an “I” for intervention or a “C” for control were printed and put in sealed envelopes. After an eligible participant had agreed to participate in the study, a home visit was scheduled. The participant signed the informed consent form and filled out the baseline questionnaire. The researcher selected an envelope from the batch and opened it and subsequently install the Activity Coach app or the sham version onto the participant’s Optilogg without informing them about the allocation.

### Blinding

This was a single-blinded, parallel pilot RCT. Every participant already had the Optilogg system at home and at the start of the study and was randomized to either the control or the intervention group at the beginning, at which point the sham version or the actual Activity Coach app was installed on their Optilogg without them knowing which version they received. The participants were not informed of what arm they were randomized to until the study was over and all data collected. The person who entered and registered all the data was also blinded to the allocation, but the investigators knew which group each participant was allocated to.

### Ethical Considerations

Ethics approval was obtained from Sweden’s Ethical Review Board (Dnr 2021-05366-01; approval date: November 17, 2021), with the amendment regarding the recruitment procedure also being approved (Dnr 2023-00799-02; approval date: February 19, 2023). All recruited participants signed and submitted their informed consent to participate in writing.

### Statistical Analysis

Continuous variables, such as age and steps, were analyzed using the Student *t* test, and categorical variables were analyzed using the chi-square test. For subjectively graded goals, a mean value was computed from the 2 answers, and the Student *t* test was performed to test the differences between groups.

To test the differences in ordinal variables, such as self-reported physical activity and exercise, as well as nonnormally distributed data, such as the scaled outputs of the KCCQ, the Mann-Whitney U nonparametric test was performed. *P*<.05 was considered statistically significant.

All statistical calculations were computed in SPSS Statistics v.29 (IBM Corporation). All statistical tests performed on outcome data were performed without power calculations and were interpreted accordingly.

## Results

### Population

**Figure 1:**
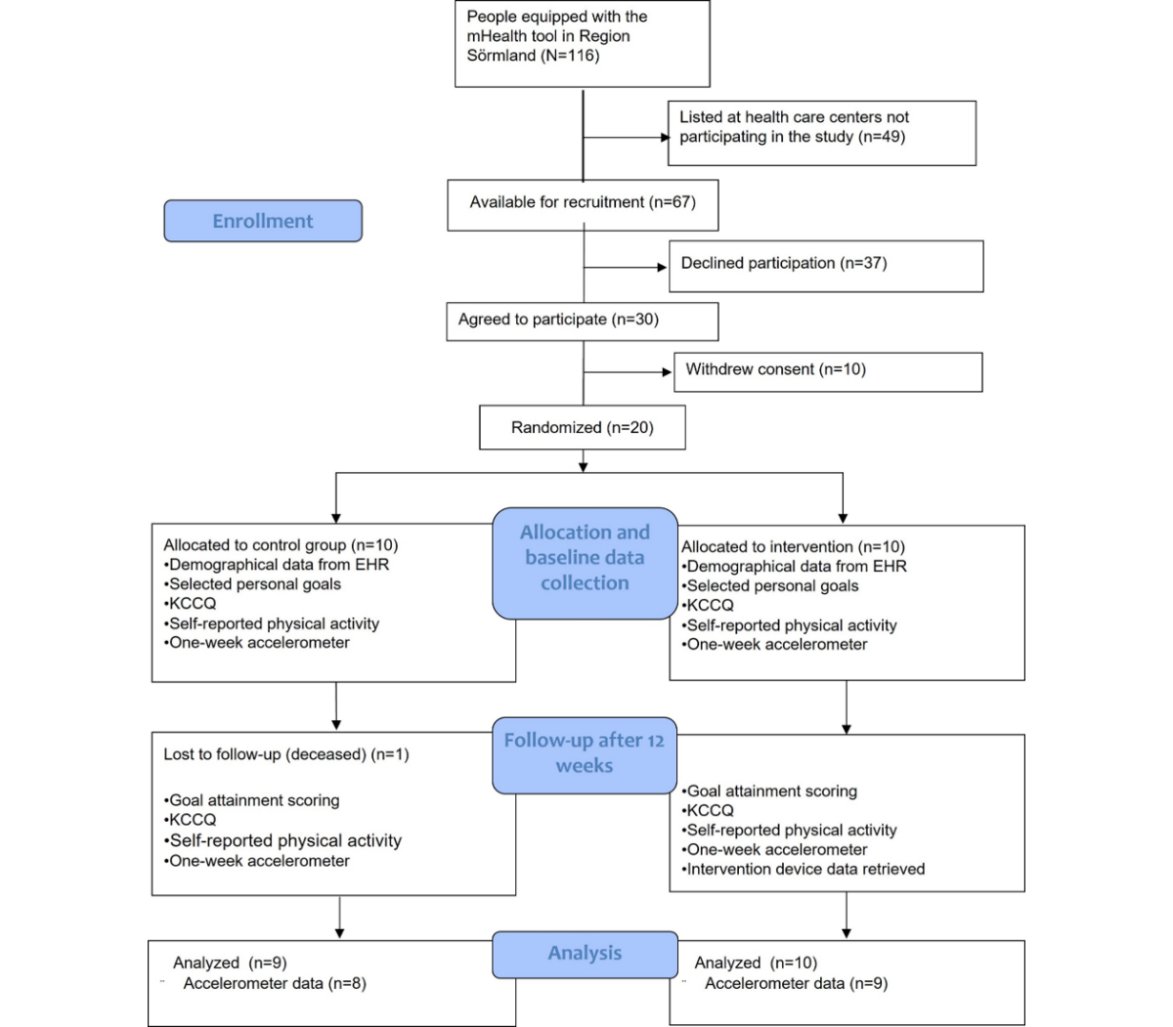
Participant flow and study design of the pilot RCT. KCCQ - Kansas City Cardiomyopathy Questionnaire, EHR – electronic health records.

The participant selection flowchart, along with the study design, is illustrated in Figure 1.

In total, 116 people were using the mHealth tool as part of their regular self-care at the start of this study. These 116 potential participants were listed at 11 different primary health care centers, 5 (45%) of which agreed to participate in the study. At these 5 health care centers, 67 (58%) of the 116 people were listed and were contacted and asked to participate. Participants were recruited between September 13, 2022, and March 25, 2023.

### Baseline Data

The participants’ baseline demographical data are summarized in Table 2. No significant differences between the 2 study arms were detected.

**Table 2:** Baseline data, including demographics, etiology, comorbidities, and pharmacological treatment, for study participants.

Characteristics			Control group (n=10)		Intervention group (n=10)		Total (N=20)		
Age (years), mean (SD)			77 (5)		78 (9)		78 (7)		
Females, n (%)			4 (40)		4 (40)		8 (40)		
**HF^a^ type, n (%)**									
	HFrEF^b^	4 (40)		3 (30)		7 (35)			
	HFmrEF^c^	1 (10)		4 (40)		5 (25)			
	HFpEF^d^	5 (50)		3 (30)		8 (40)			
**NYHA^e^ class, n (%)**									
	NYHA II	4 (40)		6 (60)		10 (50)			
	NYHA III	6 (60)		4 (40)		10 (50)			
**Comorbidities, n (%)**									
	Diabetes	3 (30)		2 (20)		5 (25)			
	COPD^f^	3 (30)		2 (20)		5 (25)			
	Hypertension	4 (40)		6 (60)		10 (50)			
	Atrial fibrillation	3 (30)		5 (50)		8 (40)			
	Kidney disease	2 (20)		3 (30)		5 (25)			
	ACEi^g^/ARB^h^/ARNI^i^	8 (80)		10 (100)		18 (90)			
	Beta blocker	7 (70)		10 (100)		17 (85)			
	MRA^j^	1 (10)		2 (20)		3 (15)			
	SGLT2i^k^	2 (20)		5 (50)		7 (35)			
	Physically inactive	7 (70)		8 (80)		15 (75)			

^a^HF: heart failure.

^b^HFrEF: heart failure with reduced ejection fraction.

^c^HFmrEF: heart failure with mildly reduced ejection fraction.

^d^HFpEF: heart failure with preserved ejection fraction.

^e^NYHA: New York Heart Association.

^f^COPD: chronic obstructive pulmonary disease.

^g^ACEi: angiotensin-converting enzyme inhibitor.

^h^ARB: angiotensin receptor blocker.

^i^ARNI: angiotensin receptor blocker neprilysin inhibitor.

^j^MRA: mineralocorticoid receptor antagonist.

^k^SGLT2i: sodium glucose cotransporter 2 inhibitor.

### Numbers Analyzed

Of the 20 participants recruited, 1 (5%) participant in the control group died during the study. Another participant in the control group and 1 (5%) in the intervention group did not meet the requirements of wear time for their accelerometer data to be included in the analyses. Hence, accelerometer-based outputs were available for 8 (80%) participants in the control group and 9 (90%) participants in the intervention group. All questionnaire-based data were available for 9 (90%) participants in the control group and 10 (100%) participants in the intervention group.

### Research Question 1: Recruitment

Of the 67 participants with HF whom we could contact, 30 (45%) agreed to participate, but 10 (33%) withdrew their consent before being formally recruited and signing the informed consent form. Most did not provide a reason, but 1 (10%) reported a spouse who had gotten sick, and another had hurt himself and did not wish to participate any longer.

Finally, 20 (30%) of the 67 potential participants were recruited and could be randomized to the control group or the intervention group. However, had the amendment to also include people who were assessed to be physically active not been made, only 15 (22%) people would have been recruited and randomized.

### Research Question 2: Implementation Fidelity

The daily adherence to registering physical activity in the Activity Coach app was 69% (range 24%-97%), and weekly adherence was 88% (range 58%-100%). On average, participants in the intervention group registered 323 (range 66-422) minutes per week. The lowest weekly adherence was 50%. In addition, 40% (n=8) of the participants had a daily adherence exceeding 90%, while 40% (n=8) had a weekly adherence of 100%. Furthermore, 9 (90%) of the 10 participants equipped with the Activity Coach app chose to enable the goal-setting functionality. The median weekly goal achievement was 83% (range 61%-96%).

### Research Question 3: Outcomes

#### Subjective Goal Attainment

All 10 prespecified goals were selected by at least 1 (5%) study participant. The first goal selected was distributed among the first 5 goals (see Table 1), whereas the choices for the second goal were distributed over 9 of the 10 options, with the most frequently selected goa being reduced shortness of breath. In the control group, 8 (80%) participants experienced overall worsening and 1 (10%) reported improvement, whereas in the intervention group, 7 (70%) participants reported improvement, 2 (20%) reported no difference, and 1 (10%) reported overall worsening.

**Figure 2:**
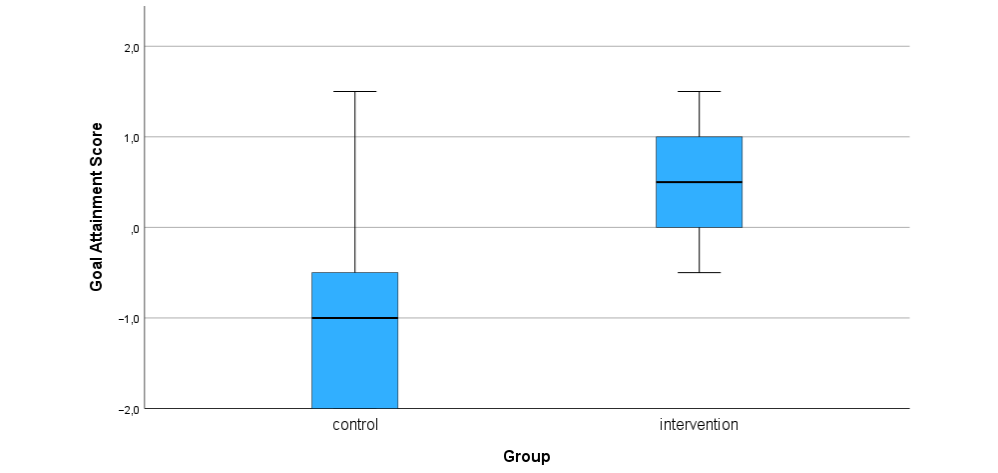
Goal attainment score by group, calculated as the mean (SD) value for the 2 selected goals.

The mean goal attainment score for the 2 groups was –1.0 (SD 1.1) for the control group versus 0.6 (SD 0.6) for the intervention group (*P*=.001). This is illustrated in Figure 2.

#### Health-Related Quality of Life

**Figure 3:**
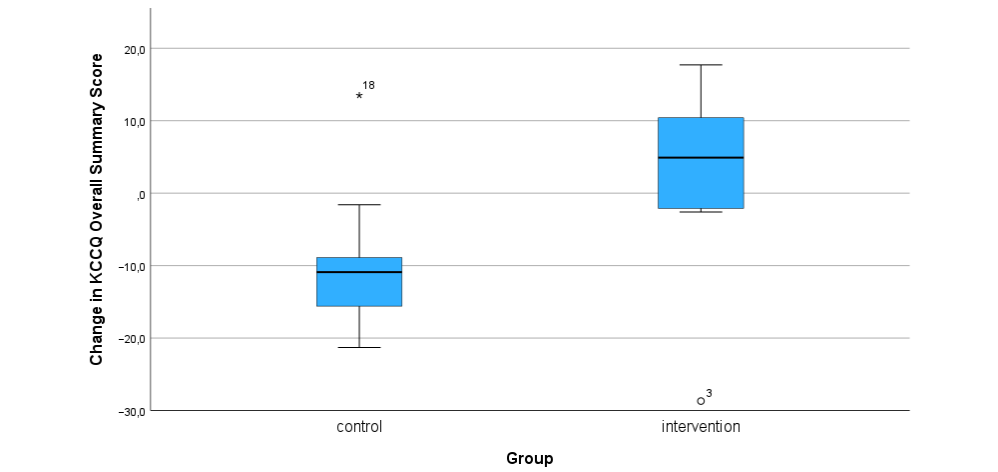
Change in the KCCQ overall summary score by group at follow-up after 12 weeks. KCCQ: Kansas City Cardiomyopathy Questionnaire.

In the control group the mean KCCQ summary score was 59 (SD 25) at baseline compared to 67 (SD 22) in the intervention group (*P*=0.6). At follow-up, the scores were 54 (SD 25) and 70 (SD 21) for the control and the intervention group, respectively (*P*=.18). ANCOVA to control for baseline differences yielded *P*=.04. The mean change in the overall summary score was –9 (SD 10) in the control group versus 3 (SD 13) in the intervention group (*P*=.03) and is illustrated in Figure 3, while the other KCCQ subscales are presented in Table 3.

**Table 3:** KCCQ^a^ output (summary score and subdimensions) at baseline and follow-up.

Subdimensions and time		Control group score, mean (SD)	Intervention group score, mean (SD)	*P* value
**Physical limitation**				
	Baseline	57 (26)	65 (28)	.46
	Follow-up	45 (27)	71 (20)	.04
**Symptom frequency**				
	Baseline	57 (33)	67 (26)	.38
	Follow-up	56 (26)	66 (30)	.29
**QoL^b^**				
	Baseline	60 (19)	67 (19)	.62
	Follow-up	63 (26)	68 (21)	.74
**Social limitation**				
	Baseline	61 (33)	69 (31)	.62
	Follow-up	54 (30)	74 (26)	.15
**Summary score**				
	Baseline	59 (25)	67 (22)	.55
	Follow-up	54 (25)	70 (21)	.18

^a^KCCQ: Kansas City Cardiomyopathy Questionnaire.

^b^QoL: quality of life.

#### Objective Physical Activity

The accelerometer-derived data are summarized in Table 4. Participants spent, on average, 76% (SD 7%) of the time in a sedentary state at baseline (control group: mean 79%, SD 6%; intervention group: mean 74%, SD 8%; *P*=.20), which at follow-up was 74% (SD 10%) of the time (control group: mean 78%, SD 8%; intervention group: mean 71%, SD 12%; *P*=.22). The decrease was mean –2.6% (SD 7%) in the intervention group versus –0.91% (SD 5%) in the control group (*P*=.59).

The average length of sedentary bouts at baseline was 408 (SD 87) minutes (control group: mean 431, SD 72 minutes; intervention group: mean 392, SD 96 minutes; *P*=.38) based on all the data recorded during the week of wearing the accelerometer. Over the course of the study, the average length of sedentary bouts increased by 27 minutes to 458 (SD 84) minutes in the control group and decreased by 0.70 minutes to 391 (SD 117) minutes in the intervention group (*P*=.22).

On average, participants walked 2735 (SD 2481) steps per day at baseline (control group: mean 2058, SD 1356 steps; intervention group: mean 3210, SD 3019 steps; *P*=.36). The average number of steps per day increased by 861 to 2919 (SD 2303) steps in the control group and by 322 to 3532 (SD 3208) steps in the intervention group (*P*=.67). The time spent in light physical activity (LPA) increased by 8 minutes in the control group and by 42 minutes in the intervention group (*P*=.07). Moderate-to-vigorous physical activity (MVPA) and LPA data are included in Table 4.

**Table 4:** Overview of objectively measured physical activity obtained from the accelerometer at baseline and 12-week follow-up.

Data items and time		Control group, mean (SD)	Intervention group, mean (SD)	*P* value
**Sedentary behavior (%)**				
	Baseline	79 (6)	74 (8)	.33
	Follow-up	78 (8)	71 (12)	.33
**Average length of sedentary bouts (minutes)**				
	Baseline	431 (72)	392 (96)	.56
	Follow-up	458 (84)	391 (117)	.14
**Steps per day, n**				
	Baseline	2058 (1356)	3210 (3019)	.33
	Follow-up	2919 (2303)	3532 (3208)	.70
**LPA^a^ (minutes)**				
	Baseline	138 (47)	169 (52)	.27
	Follow-up	146 (46)	207 (80)	.07
**MVPA^b^ (minutes)**				
	Baseline	15 (11)	31 (35)	.54
	Follow-up	21 (24)	33 (42)	.96

^a^LPA: light physical activity.

^b^MVPA: moderate-to-vigorous physical activity.

### Ancillary Analyses

Since the original trial design was modified to also include people who were not physically inactive, ancillary analyses were performed to study what impact, if any, that had on the data. No significant differences appeared. The differences are listed in Table 5.

**Figure 4:**
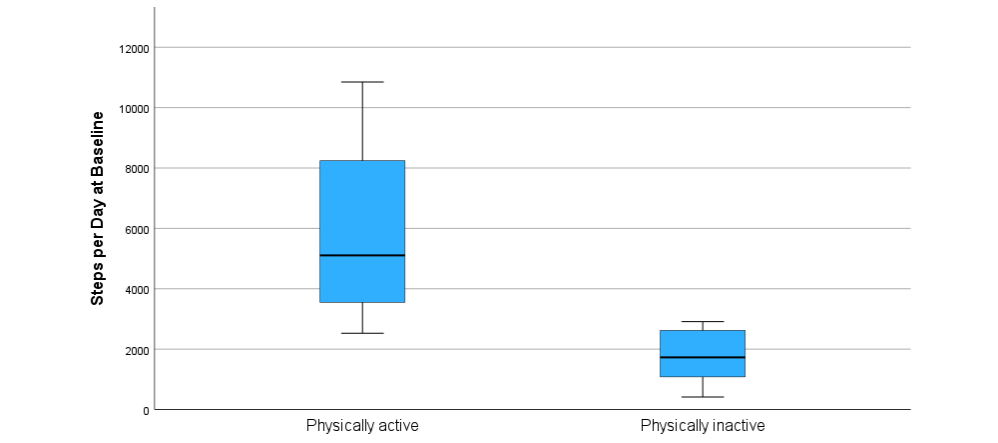
Accelerometer-measured steps per day for physically active versus physically inactive participants.

A comparison of the steps per day for the physically active versus physically inactive participants is shown in Figure 4.

**Table 5:** Comparison of objectively measured physical activity between groups with the original data set and the data set including only participants classified as physically inactive (n=15, 75%).

Data items and time		Control group, mean (SD)		Intervention group, mean (SD)
**Steps per day at baseline, n**				
	Original data set	2058 (1356)	3210 (3019)	
	Physically inactive	1459 (814)	1952 (856)	
**Steps per day at follow-up, n**				
	Original data set	2919 (2303)	3532 (3208)	
	Physically inactive	2194 (1943)	2218 (1139)	
**Average length of sedentary bouts at baseline (minutes)**				
	Original data set	431 (72)	392 (96)	
	Physically inactive	455 (71)	406 (99)	
**Average length of sedentary bouts at follow-up (minutes)**				
	Original data set	458 (84)	391 (117)	
	Physically inactive	495 (55)	409 (119)	

## Discussion

### Principal Findings

The results of this study show that it may be challenging to recruit physically inactive older adults with HF to participate in an mHealth study, but there might be benefits in using mHealth to support this population in achieving goals relating to physical activity and reduce sedentary behavior.

#### Recruitment

It is known to be challenging to recruit older people with severe illness for studies evaluating digital health technologies [27]. It seems reasonable that seeking to recruit physically inactive people makes it even more likely that they decline participation. Only 22% of the people contacted for this study were successfully recruited and randomized, although 45% initially consented to participate, but every third person withdrew consent prior to starting the trial. It might, therefore, be appropriate to investigate whether something in the recruitment process could be improved (eg, the information material or striving to initiate the trial at the same time as collecting the consent). Reasons given for withdrawal were typically negative life events (eg, having been hospitalized, having received a new diagnosis, or having a spouse whose health had deteriorated). The withdrawal rate was lower in patients who were recruited via telephone versus via mail.

The recruitment rate in this study of 22% was low compared to other studies, where the corresponding rates ranged from 70% to 90%, although these numbers come from studies in which participants were recruited in the in-patient setting [28,29]. It should also be noted that aside from the 1 participant who died, there was no attrition during the study, and all participants who started also finished the study.

Based on our experiences from this study, we conclude that researchers must be active and may have to call up each possible participant and that merely sending out letters via regular mail is likely insufficient.

#### Implementation Fidelity

To be able to judge the quality of a study and to properly replicate it, some reporting of intervention fidelity is required, and examples of aspects of intervention fidelity to report are intervention design and intervention delivery or receipt of the intervention [30]. The intervention design has been published elsewhere [9], and a qualitative study to investigate user experiences was beyond the scope of this study. An acceptable intervention delivery ensures that results from the intervention are generalizable [31]. Delivery of the intervention can be reported as intervention adherence [18], which is what we reported in this manuscript. Other mHealth studies have used 60% adherence as a cutoff point for acceptable level of use [17,32], and that level was also adopted in the development of the Activity Coach app [9]. In this study, the median adherence was 69%, which is higher than what is normally reported for mHealth interventions [19] and also higher than that in the Activity Coach feasibility study, suggesting an improvement in usability following the final adjustments made to the Activity Coach app after the feasibility study [9]. The 2022 systematic review by Jakob et al [19] concluded that factors that drive adherence are tailoring of content to the user and gamification, both of which are present in the Activity Coach app and may explain the better-than-average adherence. What level of adherence to an intervention is acceptable should depend on which intervention is being studied. The current guidelines on physical activity from the World Health Organization (WHO) for the general population [33], as well as guidelines for people with HF [34], make recommendations on a weekly basis, so it may be reasonable to also assess adherence to a tool supporting physical activity on a weekly basis; in this study, weekly adherence to the Activity Coach app was 88%, which is similar to other rates in the published literature, which ranged from 55% to 80%, depending on the definition of adherence [29,35-39].

#### Outcomes

##### Plausibility of Outcomes

Questionnaires were completed by 19 of the 20 participants, and system (Activity Coach) data from all 10 participants in the intervention group and accelerometer data from 17 of the 20 participants passed wear time scrutiny. The use of a prespecified list of personal goals to choose from, which was a learning from a feasibility study [9], worked well, and each participant successfully selected 2 goals and could grade them at follow-up. All data were analyzable and of acceptable quality.

##### Limited Efficacy Testing

Goal attainment is an important part of person-centered care [40,41] and is also more responsive to change than other measures [42]. The 5-point scale we used is considered the norm when using goal attainment as an outcome [43,44]. There was a significant difference in goal attainment between the 2 groups, and although there was a subtle improvement in the intervention group, there was a more pronounced worsening in the control group.

It is difficult to assess the clinical importance of a goal attainment scaling outcome, but a 25% shift on an ordinal scale has previously been used as a clinically meaningful change [41,45]. The shift in this study was of a magnitude of 32% of the used scale. In the light of improvements in the HR-QoL, this might imply that there is a clinically meaningful effect.

The KCCQ results mirrored the subjective goal attainment results, in that the KCCQ summary score showed a 12-point difference between the 2 groups, expressed as a 5% improvement in the intervention group and a 15% worsening in the control group. This difference is of a similar size as effects reported from another mHealth intervention aimed at increasing physical activity, which reported a 6-point difference, also at 12-week follow-up [46]. Other telehealth interventions, albeit not strict mHealth, have reported smaller or no improvements in the KCCQ score [47-50]. The difference between the 2 groups at baseline in the summary score was large, albeit nonsignificant, and when that difference was corrected for, the difference between the 2 groups at follow-up also became significant.

Looking at the subscales, the physical limitation dimension was the subdomain with the largest effect, where the intervention group at follow-up had a 37% higher score. A change in that subscale exceeding 8.33 points has been identified as the minimal clinically important difference for improvement and a decrease of 4.17 or more indicates deterioration [51]. In this study, the score of the intervention group increased by 6 points and that of the control group decreased by 12 points, which suggests a clinically meaningful deterioration in the control group.

There was a significant difference of 4135 in the steps per day between the physically inactive versus active participants, which needs to be considered when interpreting the results from the objective measurements of physical activity. The change in the time spent in sedentary behavior was of a magnitude similar to that in other reports on physical activity interventions [52]. The differences between the 2 study arms were more pronounced after removing the physically active participants, with an increase of 40 minutes in the control group compared to an increase of 3 minutes in the intervention group.

Longer sedentary bouts are associated with poor health outcomes [53-55]. For these effects to manifest into outcomes, the reduction in sedentary time needs to be shifted to MVPA according to Rossen et al [56], which was, however, disputed by Diaz et al [57]. There is still a knowledge gap regarding the impact of the sedentary bout length and the effects of changing it [58].

There was no significant difference in the steps per day between the 2 groups, neither at baseline nor at follow-up, although the number of steps per day increased more in the control group (861 steps) compared to the intervention group (322 steps). The increase in steps in the control group is large enough to potentially be clinically meaningful [59], although successful interventions aimed at increasing the step count in chronically ill older adults usually report larger effects (eg, mean ~2000, SD 500, more steps per day or above) [28,60,61]. It is not possible to say whether this effect on the control group was a consequence of the small sample size or external factors. Perhaps there is a connection between more steps per day and longer sedentary bouts. Other research has also proven the positive effects of mHealth interventions on physical activity outcomes [62].

Overall, it appears that the Activity Coach app does not improve the outcomes studied, but it may offer protection against deterioration, as the control group experienced worsened situations relating to their individual goals, a significantly worse HR-QoL, and a trend toward increased length of sedentary bouts. The Activity Coach app does not necessary tell the user to go out and walk, but it focuses more on subtle suggestions, such as standing up a couple of times or doing leg kicks during television commercials, shifting the balance between the feet, doing calf raises while doing the dishes, or using an exercise band. Assuming this is adhered to, it would explain a perceived increase in exercise and improvements in the HR-QoL and goal attainment, without necessarily showing up on the step counts. Possibly, it is manifested in the shorter average lengths of sedentary bouts.

Combining the recruitment rate with the effect sizes on the outcomes measured showed that a future efficacy study designed in the same way, with the primary endpoint being either the KCCQ summary score or sedentary time, needs to contact approximately 250-350 people to be sufficiently powerful.

### Limitations

The original study design was modified, and as seen in the ancillary analyses, there were significant differences in daily physical activity between physically active and physically inactive participants, which makes the results more difficult to interpret.

Fewer participants than desired were included, and even though the study was not designed based on specific power calculations, a larger included population would have provided better grounds for designing an efficacy trial, a more well-grounded sample size estimation, and improved generalizability.

### Conclusion

The recruitment rate of this study, where older adults with HF were to evaluate an mHealth tool, was 22%, which is a low rate. An active recruitment process is advised if a future efficacy study is to be performed. Adherence to the Activity Coach intervention was better than average at 69%. Deterioration in health and the QoL may be attenuated by the Activity Coach app, possibly by increasing certain aspects of physical activity and by reducing the length of sedentary bouts.

## Appendix

Multimedia Appendix 1 CONSORT-eHEALTH checklist (V 1.6.1).

## Abbreviations

EHR: electronic health record
GUI: graphical user interface
HF: heart failure
HR-QoL: health-related quality of life
KCCQ: Kansas City Cardiomyopathy Questionnaire
LPA: light physical activity
MRC: Medical Research Council
MVPA: moderate-to-vigorous physical activity
QoL: quality of life
RCT: randomized controlled trial

## Data Availability

The data sets generated and analyzed during this study are available from the corresponding author upon reasonable request.
